# Extracellular vesicle–associated and non–vesicle-associated microRNAs for early breast cancer detection: a systematic review

**DOI:** 10.3389/fgene.2026.1716692

**Published:** 2026-05-12

**Authors:** Jessica Daniela Romero Urrego, Carlos Alberto Parra-López, Mario Arturo Isaza‐Ruget, Yovana Pacheco

**Affiliations:** 1 Fundación Universitaria Sanitas, Bogotá, Colombia; 2 Facultad de Ciencias, Instituto de Biotecnología, Universidad Nacional de Colombia, Bogotá, Colombia; 3 Departamento de Microbiología, Facultad de Medicina, Grupo de Investigación en Inmunología y Medicina Traslacional, Universidad Nacional de Colombia, Bogotá, Colombia; 4 Laboratorio Clínico y de Patología, Clínica Colsanitas, Bogotá, Colombia

**Keywords:** biomarkers, blood-based biomarkers, breast cancer, circulating microRNAs (miRNAs), early diagnosis, exosomal microRNAs, extracellular vesicles, liquid biopsy

## Abstract

Breast cancer remains a leading cause of morbidity and mortality worldwide, with survival outcomes strongly dependent on early detection. Conventional mammography, while widely adopted, faces limitations in sensitivity—particularly in dense breast tissue—and is not without invasiveness. Circulating microRNAs (miRNAs), both exosomal and non-exosomal, have emerged as promising non-invasive biomarkers due to their stability in biofluids and cancer-associated dysregulation. This systematic review evaluated studies published between 2016 and 2025 assessing the diagnostic performance of blood-derived miRNAs for early breast cancer detection. Several individual miRNAs, including miR-21-5p and miR-200c, as well as panels such as those comprising miR-106a-3p, miR-106a-5p, miR-20b-5p, and miR-92a-2-5p, demonstrated high diagnostic accuracy (Area Under the Curve (AUC) > 0.9) in distinguishing breast cancer patients from healthy controls. Despite challenges related to tumor heterogeneity and pre-analytical standardization, integrating circulating miRNA biomarkers with current imaging modalities holds significant potential to enhance early detection and support personalized management strategies.

## 1 Introduction

Breast cancer is the most common malignancy worldwide, with an estimated 2.3 million cases diagnosed and 670,000 deaths reported in 2022 (World Health Organization, 2025). In Colombia, breast cancer is the most frequently diagnosed cancer and the leading cause of cancer mortality among women, with an age-standardized incidence rate of approximately 50.7 per 100 000 females (Bray et al., 2024). Mammography remains the primary modality for breast cancer screening and early detection, recommended by numerous international and national guidelines, including the Comprehensive Care Guide for the early detection, comprehensive care, follow-up, and rehabilitation of patients diagnosed with breast cancer in Colombia. For instance, in women under 50 years of age, denser breast tissue can complicate the identification of malignancy (Giaquinto et al., 2024). More importantly, the temporal aspect of diagnosis is critical: early detection of breast cancer is essential, as the 5-year survival rate for patients diagnosed at a localized stage can reach approximately 99%, whereas it decreases dramatically to around 32% in cases diagnosed at the distant metastatic stage. Furthermore, current invasive procedures such as biopsies cause pain and stress in patients, underscoring the critical need for complementary non-invasive diagnostic methods (Giaquinto et al., 2024).

In this context, microRNAs (miRNAs) have emerged as promising biomarkers due to their involvement in gene expression regulation (Hannafon et al., 2016; Gao B. et al., 2017; Kumar et al., 2020). MiRNAs are small, non-coding RNA molecules that function at the post-transcriptional level, either by facilitating the degradation or inhibiting the translation of specific messenger RNAs (mRNAs) (Li et al., 2018).

They are produced from longer precursors through a sophisticated cleavage process in the nucleus and cytoplasm, adopting a distinctive hairpin configuration during their biogenesis. Their regulation is complex, involving various transcriptional and post-transcriptional factors, which enables them to orchestrate a wide range of cellular processes. Once mature, miRNAs are remarkably stable in various biological fluids such as blood, urine, and cerebrospinal fluid, with a half-life that can range from hours to days, making them ideal candidates for diagnostic biomarkers (Zou et al., 2021).

The utility of miRNAs as biomarkers extends beyond breast cancer; they have been extensively investigated and validated in the diagnosis and prognosis of other cancer types, as prostate and ovarian cancers, among others, where their altered expression profiles correlate with disease progression and treatment response (Khan et al., 2019; Kumar et al., 2020; Ozawa et al., 2020). Specifically in breast cancer, it has been identified that miRNA expression is dysregulated in breast cancer patients compared to control patients (Asgari and Rezaie, 2022).

These miRNAs can be found either circulating freely in the bloodstream (Zou et al., 2021) or encapsulated within extracellular vesicles (EVs), including exosomes (Ozawa et al., 2020), which protects them from degradation and broadens their potential as diagnostic tools. It is important to note that while this review focuses on the early diagnosis of breast cancer, previous studies on miRNAs in this pathology have indeed been conducted. However, many of these studies have concentrated on monitoring disease progression or treatment response. Therefore, continued research is fundamental to further understanding the role of miRNAs in early detection and optimizing their clinical application at this crucial stage.

In this context, this systematic review aims to summarize current evidence on circulating miRNAs, including those associated with EVs and non-vesicle-associated fractions, as diagnostic biomarkers for breast cancer, with a particular focus on their potential applicability in early detection.

### 1.1 Biogenesis and function of miRNAs

MiRNAs are small endogenous non-coding RNA molecules, typically 18–25 nucleotides in length, that regulate gene expression at the post-transcriptional level. They exert their regulatory function primarily through partial sequence complementarity to sequences located in the 3′-untranslated region (3′-UTR) of target mRNAs. After incorporation into the RNA-induced silencing complex (RISC), miRNAs guide this complex to target transcripts, promoting translational repression or facilitating mRNA degradation through transcript destabilization. Through this regulatory process, miRNAs participate in the fine-tuning of a wide range of biological and pathological processes, including cell proliferation, apoptosis, differentiation, metabolism, inflammation, and stress responses. Dysregulation of miRNA expression has been widely implicated in cancer initiation, progression, metabolic reprogramming, and metastatic dissemination, highlighting their dual role as oncogenic or tumor-suppressive regulators depending on cellular context and target genes (Keshavarzmotamed et al., 2023; Naeimzadeh et al., 2023; Chen et al., 2024; Qiong et al., 2024; Hu et al., 2025).

MiRNA biogenesis begins in the cell nucleus, where RNA polymerases II and III synthesize the primary miRNA transcript (pri-miRNA), which contains a characteristic hairpin structure. This pri-miRNA is processed by the Drosha-DGCR8 microprocessor complex, which cleaves it to generate a precursor miRNA (pre-miRNA) that is subsequently exported to the cytoplasm via the exportin-5–Ran-GTP complex. Once in the cytoplasm, the pre-miRNA is further cleaved by the Dicer (RNase III) complex together with the Trans-Activator RNA Binding Protein (TRBP), generating a mature miRNA duplex by removing the hairpin loop.

**FIGURE 1:**
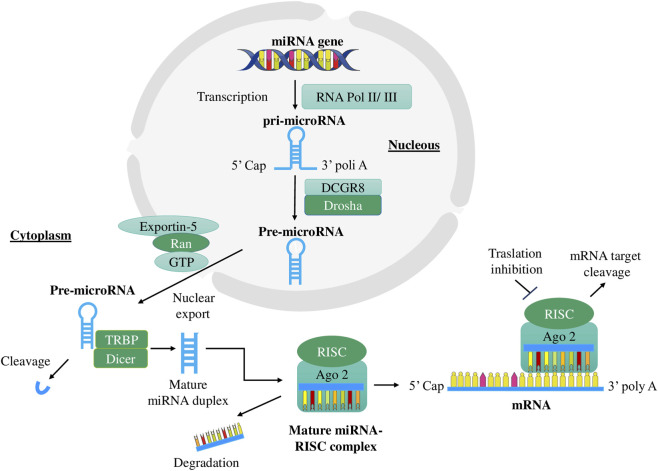
Canonical biogenesis and mechanism of action of microRNAs. MicroRNAs (miRNAs) are transcribed by RNA polymerase II or III as primary miRNAs (pri-miRNAs) and processed in the nucleus by the Drosha–DGCR8 complex into precursor miRNAs (pre-miRNAs). Pre-miRNAs are exported to the cytoplasm by Exportin-5 in a Ran–GTP–dependent manner and further cleaved by Dicer in association with TRBP to generate a ∼22-nt miRNA duplex. The guide strand is incorporated into the RNA-induced silencing complex (RISC) with Argonaute-2 (Ago2), while the passenger strand is degraded. The mature miRNA–RISC complex regulates gene expression through sequence-specific binding to target mRNAs, resulting in translational repression or mRNA degradation. Abbreviations: miRNA, microRNA; pri-miRNA, primary microRNA; pre-miRNA, precursor microRNA; DGCR8, DiGeorge syndrome critical region 8; TRBP, TAR RNA-binding protein; Ago2, Argonaute 2; RISC, RNA-induced silencing complex; UTR, untranslated region.

In the conventional miRNA maturation pathway, known as the canonical pathway, the miRNA duplex composed of a functional strand and a passenger strand is incorporated together with Argonaute 2 (Ago2) proteins into the pre-RISC complex (pre-miRNA-induced silencing complex) (Khan et al., 2019). Within the Argonaute proteins, the duplex is unwound, the guide strand is selected, and the passenger strand is discarded. This process results in the formation of the mature miRNA–RNA-induced silencing complex (RISC), which regulates gene expression by binding to the 3′-UTR of target mRNAs, leading to translational repression or transcript degradation (Figure 1) (Kumar et al., 2020).

In this duplex, the guide strand generally corresponds to the 5p miRNA, while the passenger strand corresponds to the 3p miRNA. Although the passenger strand was initially thought to be discarded, accumulating evidence indicates that it can also be functionally active. Notably, in cancer and other pathological conditions, the relative abundance and functional roles of the 5p and 3p miRNA strands can be reversed. Importantly, the 5p and 3p miRNA strands derived from the same precursor may regulate distinct sets of target mRNAs, thereby contributing to different biological effects. Once mature and incorporated into the RISC, miRNAs exhibit remarkable stability, with half-lives ranging from hours to several days in various biological fluids, which contributes to their utility as biomarkers.

The diagram outlines sequential steps: transcription of miRNA genes in the nucleus, processing of pri-miRNA into pre-miRNA, export to the cytoplasm, and cleavage by the Dicer–TAR RNA-binding protein (TRBP) complex to generate a mature miRNA duplex. The guide strand is incorporated into the RISC, where it regulates gene expression by repressing translation or promoting degradation of target mRNAs. Image adapted from (Khan et al., 2019; Kumar et al., 2020).

MiRNAs involved in cancer are broadly classified into oncogenic miRNAs (oncomiRs), which are typically overexpressed and promote tumorigenesis, and tumor-suppressor miRNAs, which are frequently downregulated and normally inhibit oncogenic pathway (Kumar et al., 2023). Dysregulation of these miRNAs contributes to cancer development and progression by influencing key processes such as apoptosis, cell cycle control, and major signaling pathways (Kumar et al., 2023). Owing to their differential expression between breast cancer patients and healthy individuals (Hannafon et al., 2016; Gao B. et al., 2017; Pan et al., 2022; Kumar et al., 2023; Turkoglu et al., 2024) and their stability in various biological fluids such as blood (Zou et al., 2021), miRNAs represent powerful candidates for detecting breast cancer across different disease stages (Zou et al., 2021).

### 1.2 Differential expression in breast cancer

For example, elevated levels of circulating miRNAs such as miR-1246, miR-122, and miR-21 have been detected in plasma and serum samples from breast cancer patients, while exosomal let-7a has been specifically reported in EVs derived from patient biofluids. These miRNAs have demonstrated the capacity to influence nearby normal cells and distant tissues, favoring the development of more aggressive tumors with increased proliferation. Among them, miR-21 stands out due to its strong association with metastasis and advanced disease stages, acting as a promoter of tumor progression (Hannafon et al., 2016). Likewise, miR-7641 has also been associated with metastatic disease. Its significantly elevated levels in the plasma of patients with metastasis suggest a potential role in promoting cancer cell growth (Shen et al., 2021). These findings reinforce the concept that miRNAs are critical regulators in breast cancer progression.

In this context, miRNAs have been extensively investigated because of their association with metastatic processes. For example, serum and exosomal miR-370-3p have been reported to be overexpressed in breast cancer patients and to correlate with lymphatic and nodal metastasis. Mechanistically, miR-370-3p promotes breast cancer proliferation and metastasis by inhibiting Fibulin-5 (FBLN5) expression and activating the NF-κB signaling pathway (Mao et al., 2021).

Likewise, miR-24-3p showed significant overexpression in plasma from women with breast cancer, especially in those in stages I–III who subsequently developed metastasis, suggesting a key role in metastatic progression (Khodadadi-Jamayran et al., 2018).

At the level of therapy resistance, overexpression of exosomal miR-181b-5p in breast cancer cells *in vitro* was shown to reduce p53/p21 levels and inhibit G1 phase arrest and doxorubicin-induced senescence by suppressing BCL-2–associated transcription factor 1 (BCLAF1) expression (Zhao et al., 2023).

It is crucial to note that the reduction of p53/p21 levels is critical, as p53 is a well-known tumor suppressor gene that plays a fundamental role in cell cycle arrest and apoptosis. Therefore, its decrease promotes the survival and proliferation of cancer cells and contributes to drug resistance. Additionally, the authors demonstrated that treatment with exosomal miR-181b-5p inhibitors *in vivo* improved tumor control and reversed drug resistance by increasing BCLAF1 expression (Zhao et al., 2023). In addition, breast cancer cell-derived exosomes were found to promote bone metastasis by transferring miR-21 to osteoclasts, which accelerates their transformation and contributes to cancer dissemination (Yuan et al., 2021).

In contrast to cancer-promoting miRNAs, there are also miRNAs that act as tumor suppressors. For example, miR-137 and miR-496 were found to be downregulated in patients with triple negative breast cancer (TNBC), and they were observed to act specifically by decreasing developmental endothelial locus-1 (Del-1) expression, which generated inhibition of cancer cell proliferation, invasion and migration, results that support their antitumor effect (Lee et al., 2021). Another important miRNA is miR-107, whose upregulation in breast cancer cells *in vitro* has been shown to suppress tumor progression, proliferation, and dissemination, primarily through the regulation of brain-derived neurotrophic factor (BDNF), a key mediator of cancer development. (Gao B. et al., 2017). Complementarily, miR-125a was significantly downregulated in breast cancer patients compared with controls (Turkoglu et al., 2024), highlighting its potential tumor-suppressive function.

The methodological quality and risk of bias of the studies included in this review were evaluated using the QUADAS-2 tool, with the results summarized in Table 1.

**TABLE 1:** Quality assessment of included diagnostic studies using the QUADAS-2 tool.

Study	Patient selection	Index test	Reference standard	Flow and timing
Exosomal hsa-miR-21-5p is a biomarker for breast cancer diagnosis (Liu et al., 2021)	High risk	High risk	Low risk	Unclear risk
Serum exosomal miR-200c is a potential diagnostic biomarker for breast cancer (Qiao et al., 2024)	High risk	High risk	Low risk	Unclear risk
Total blood exosomes in breast cancer: Potential role in crucial steps of tumorigenesis (Konoshenko et al., 2020)	High risk	High risk	Unclear risk	Unclear risk
A Breast Cancer Prediction Model Based on a Panel from Circulating Exosomal miRNAs (Pan et al., 2022)	High risk	Unclear risk	Low risk	Unclear risk
Circulating exosomal miR-363-5p inhibits lymph node metastasis by downregulating PDGFB and serves as a potential noninvasive biomarker for breast cancer (Wang et al., 2021)	High risk	Unclear risk	Low risk	Unclear risk
Detection significance of miR-3662, miR-146a, and miR-1290 in serum exosomes of breast cancer patients (Li et al., 2021)	High risk	Unclear risk	Low risk	Unclear risk
Identification of serum exosomal miR-148a as a novel prognostic biomarker for breast cancer (Li et al., 2020)	High risk	Low risk	Low risk	Low risk
Diagnostic value of circulating miR-202 in early-stage breast cancer in South Korea (Kim et al., 2020)	High risk	High risk	Low risk	Low risk
A five-miRNA panel in plasma was identified for breast cancer diagnosis (Li et al., 2019)	Moderate risk	Low risk	Low risk	Low risk
Presence of Circulating miR-145, miR-155, and miR-382 in Exosomes Isolated from Serum of Breast Cancer Patients and Healthy Donors (Gonzalez-Villasana et al., 2019)	High risk	Moderate risk	Low risk	Moderate risk
Circulating microRNAs from the miR-106a–363 cluster on chromosome X as novel diagnostic biomarkers for breast cancer (Li et al., 2018)	Moderate risk	Low risk	Low risk	Low risk
MicroRNA-155, induced by FOXP3 through transcriptional repression of BRCA1, is associated with tumor initiation in human breast cancer (Gao et al., 2017a)	High risk	Moderate-High risk	Low risk	High risk
Differential Expression of Serum Exosomal miRNAs in Breast Cancer Patients and Healthy Controls (Asgari and Rezaie, 2022)	High risk	High risk	Unclear risk	High risk
Correlative expression of exosomal miRNAs in chemotherapy resistance of triple-negative breast cancer: An observational study (Yang et al., 2024)	High risk	High risk	Low risk	High risk
Exosomal microRNA-92b Is a Diagnostic Biomarker in Breast Cancer and Targets Survival-Related MTSS1L to Promote Tumorigenesis (Kan et al., 2024)	Moderate risk	Moderate risk	Low risk	Low risk

Abbreviations: QUADAS-2, Quality Assessment of Diagnostic Accuracy Studies 2.

### 1.3 Extracellular vesicle–associated and non–vesicle-associated circulating miRNAs

Accumulating evidence indicates that miRNAs play an important role in cellular communication and various biological processes in diseases such as breast cancer, which is why they have been proposed as possible biomarkers for liquid biopsy from blood. Additionally, they are valuable because they can be easily detected in serum, plasma, or whole blood, eliminating the need for invasive procedures (Ozawa et al., 2020).

MiRNAs can be found in two main forms in the blood: as circulating non-vesicular miRNAs or associated with EVs. When circulating, these miRNAs are not truly free in the extracellular environment but are typically bound to protein complexes, such as Argonaute 2 (Ago2), high-density lipoproteins (HDL), and other RNA-binding proteins, which protect them from RNase-mediated degradation. In this non-vesicular form, circulating miRNAs emerge as promising biomarkers due to their high stability in the extracellular medium.

Alternatively, miRNAs can be encapsulated within EVs, a heterogeneous group of membrane-bound particles released by cells. Among these, exosomes represent a well-characterized EV subtype typically ranging from 50 to 150 nm in diameter and originating from the endosomal pathway. Cancer cells actively release EVs, including exosomes, into the circulation, and experimental models have demonstrated that these vesicles can be transferred from primary tumors into the bloodstream. EV-associated miRNAs, particularly those contained in exosomes, may reflect molecular characteristics of the tumor of origin, making them potentially more specific indicators of tumor presence and biological behavior (Hannafon et al., 2016).

Given these distinct forms and their unique properties, Table 2 summarizes the main advantages and limitations of EV-derived and non-vesicular (circulating) miRNAs as biomarkers, considering aspects such as stability, specificity, sensitivity, and associated extraction methods. This comparison highlights key differences, including the greater stability and biological specificity of EV-derived miRNAs and the greater accessibility and lower cost of non-vesicular circulating miRNAs.

**TABLE 2:** Comparison of EV-derived and non-vesicular miRNAs as biomarkers for cancer diagnosis.

Characteristic	EV- miRNAs	Non-EV miRNAs
Stability	High (protected by lipid membrane)	Low (susceptible to degradation)
Specificity	High (Tumor load specific)	Low (produced by multiple cells)
Sensitivity	High (high concentration in exosomes)	Moderate (sample dependent)
Extraction methods	Requires specific prior techniques for exosome extraction	Direct extraction with standard kits
Advantages	Greater stability and specificity	Easy access and lower cost
Limitations	High cost	Less stability and variability

Abbreviations: miRNA, microRNA; EV, extracellular vesicle.

MiRNAs associated with EVs, including exosomes, and those free in circulation present unique characteristics that make them useful as biomarkers for breast cancer. EV-associated miRNAs, particularly exosomal miRNAs, may offer greater specificity and stability (Hannafon et al., 2016) as they are protected by vesicular membranes, which makes them promising tools for early diagnosis and monitoring of the disease. On the other hand, circulating miRNAs have important practical advantages: their detection in serum or plasma is technically simpler and more cost-effective. The choice of one or the other type of miRNA will depend on clinical (disease stage, tumor type) and logistical (costs, available infrastructure) factors. A promising strategy may involve combining both approaches, leveraging the accessibility of circulating miRNAs and the biological specificity of exosomal miRNAs, which could offer a comprehensive approach to the development of more robust and complete biomarkers for early detection of breast cancer.

## 2 Methodology

### 2.1 Study design and reporting guidelines

**FIGURE 2:**
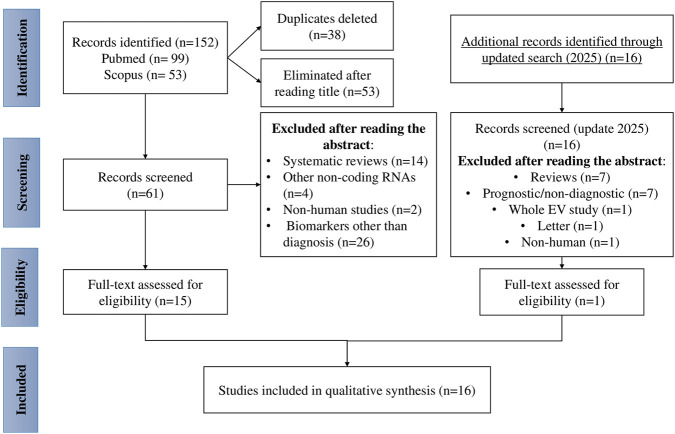
Flow diagram of the study selection process for the systematic review. The flow diagram illustrates the identification, screening, eligibility assessment, and final inclusion of studies evaluating circulating microRNAs as biomarkers for early breast cancer detection, in accordance with PRISMA guidelines. The process includes a targeted update search to identify additional eligible studies published in 2025. Abbreviation: PRISMA, Preferred Reporting Items for Systematic Reviews and Meta-Analyses.

This systematic review was conducted following the Preferred Reporting Items for Systematic Reviews and Meta-Analyses (PRISMA) guidelines (Sánchez-Serrano et al., 2022). The PRISMA flow diagram summarizing the processes of identification, screening, eligibility, and inclusion is presented in Figure 2.

### 2.2 Literature search strategy

A systematic literature search was conducted in December 2024 with an updated targeted search performed in January 2025, using the PubMed and Scopus databases. The search was limited to peer-reviewed articles with available full-text access, published between January 2016 and December 2025, and written in English.

The complete search strategy applied to both databases was as follows: (microRNA OR miRNA OR “micro RNA”) AND (blood OR serum OR plasma) AND (exosomal OR exosome OR “extracellular vesicles”) AND (“breast cancer”) AND (biomarker OR diagn*)

Search terms were adapted slightly to comply with the specific requirements of each database. No filters related to study design were initially applied in order to maximize sensitivity.

To ensure inclusion of the most recent evidence, a targeted update focused on studies published in 2025 was performed in PubMed using the same predefined search strategy. This update retrieved 16 records published in 2025. After screening, seven review articles were excluded, seven studies were excluded because they focused on prognostic applications or outcomes unrelated to early diagnosis, one study was excluded because it analyzed whole EVs rather than miRNAs as biomarkers, one study was excluded as a letter to the editor, and one non-human study using humanized mouse models was excluded. Following this process, one additional original diagnostic study published in 2025 met all inclusion criteria and was incorporated into the final qualitative synthesis.

### 2.3 Eligibility criteria

#### 2.3.1 Inclusion criteria

Studies were included if they met all of the following criteria:

- Type of study: Original research articles, including observational studies and clinical studies.
- Biomarker evaluation: Studies evaluating circulating miRNAs, either exosomal or non-exosomal, as diagnostic biomarkers for breast cancer were included. Studies with mixed-stage populations were eligible provided that their findings were relevant to diagnostic performance, including potential application to early-stage disease.
- Sample type: Studies analyzing miRNAs from blood-derived samples (serum, plasma, or isolated exosomes).
- Population: Human subjects with breast cancer, including early-stage and advanced-stage disease, provided that diagnostic performance with potential relevance for early detection was evaluated. Whenever reported, studies preferentially included treatment-naïve patients; however, studies with mixed or unspecified treatment status were retained and this limitation was considered during qualitative synthesis.

Outcome: Studies reporting diagnostic performance metrics (e.g., sensitivity, specificity, Area Under the Curve (AUC)) or differential expression relevant to early breast cancer detection.

#### 2.3.2 Exclusion criteria

##### 2.3.2.1 Studies were excluded based on the following criteria

- Publication type: Reviews, meta-analyses, letters to the editor, conference abstracts, editorials, commentaries, preprints, and case reports were excluded due to the lack of peer review and/or insufficient methodological detail.
- Biomarker type: Studies focusing on biomarkers other than miRNAs (e.g., proteins, DNA, circulating tumor cells).
- Disease type: Studies investigating cancers other than breast cancer.
- Sample origin: Studies analyzing miRNAs derived from tissues, cell lines, or non-blood biological fluids.
- Language: Articles not published in English.
- Publication date: Studies published before 2016.

### 2.4 Definition of early breast cancer

In this review, the term early breast cancer refers to disease detected at an early clinical stage, generally corresponding to stages 0–II according to the American Joint Committee on Cancer (AJCC) tumor–node–metastasis (TNM) classification when explicitly reported. However, not all included studies restricted their analyses to early-stage disease or provided detailed staging information. Several studies included mixed-stage cohorts or did not specify tumor stage. These studies were retained when they reported diagnostic performance of circulating miRNAs with potential relevance for early detection. Variations in staging definitions across studies were documented and considered during qualitative synthesis.

### 2.5 Study selection process

A total of 152 records were initially identified (99 from PubMed and 53 from Scopus). After removal of 38 duplicates, titles were screened, resulting in 61 potentially relevant articles. Following abstract screening, 46 articles were excluded due to the following reasons: systematic reviews (n = 14), studies on other non-coding RNAs (n = 4), non-human studies (n = 2), and studies not focused on diagnostic applications (n = 26). After full-text evaluation, 15 studies met all inclusion criteria and were included in the final qualitative synthesis. In addition, a targeted update search was subsequently conducted to capture studies published in 2025. One additional study met the inclusion criteria and was incorporated into the final qualitative synthesis, resulting in a total of 16 included studies.

### 2.6 Quality assessment (QUADAS-2)

The methodological quality and risk of bias of the included diagnostic studies were independently assessed using the Quality Assessment of Diagnostic Accuracy Studies-2 (QUADAS-2) tool (Whiting et al., 2011). The tool evaluates four domains: patient selection, index test, reference standard, and flow and timing, each assessed in terms of risk of bias, while the first three domains are also assessed for concerns regarding applicability. Each domain was rated as “low risk”, “high risk”, or “unclear risk” according to the signaling questions recommended by the QUADAS-2 guidelines. Discrepancies were resolved by discussion among the authors. The results of the quality assessment are summarized in Table 1.

### 2.7 MiRNA extraction and analysis techniques

The accurate detection and quantification of miRNAs is fundamental not only to understanding their role in gene expression regulation and their involvement in diseases such as breast cancer, but also to reliably assessing their potential as diagnostic biomarkers, as explored in this systematic review. This process critically depends on two key steps: miRNA extraction and subsequent analysis. Considering the methodologies employed in the studies included in this review, the most used techniques at each stage are described below, highlighting their advantages, limitations, and specific applications in breast cancer research as reported in the literature.

The methodologies described below are discussed with consideration of whether miRNAs were analyzed as circulating non-vesicular molecules or as extracellular vesicle–associated miRNAs, as these two sources differ in their pre-analytical handling, extraction strategies, and analytical requirements.

### 2.8 MiRNA extraction techniques

MiRNA extraction is a determining step that directly influences the quality and reproducibility of the results. Since circulating miRNAs are present in low concentrations and small size, it is essential to select an efficient and appropriate extraction method according to the type of sample (Hamam et al., 2017). Currently, two main approaches stand out:

#### 2.8.1 Trizol-based methods

Trizol is widely used as an initial step in miRNA extraction due to its efficiency in cell lysis and RNA stabilization. In many protocols, Trizol is not applied as a standalone method but rather as part of hybrid extraction approaches, where organic extraction is followed by column-based purification to improve RNA yield and purity. While Trizol-based protocols are cost-effective and broadly accessible, several studies have reported lower miRNA recovery and higher variability compared with fully column-based commercial kits, particularly in circulating miRNA analyses (Trakunram et al., 2019).

#### 2.8.2 Commercial kits such as miRNeasy

Commercial miRNA extraction kits commonly employ column-based purification strategies, which in some platforms are combined with an initial phenol/chloroform extraction step to enhance RNA recovery and purity. These hybrid approaches offer notable advantages, including higher yield, improved RNA quality, and reduced technical variability (Trakunram et al., 2019).

In addition, standardized protocols included in commercial kits improve reproducibility and are compatible with automated workflows. Importantly, more recent commercial platforms avoid the use of phenol/chloroform altogether and rely exclusively on silica membrane–or magnetic bead–based chemistries optimized for low-input biofluids such as serum and plasma. These phenol-free kits provide increased safety, reduced hands-on time, and consistent recovery of small RNAs, making them particularly suitable for clinical and translational circulating miRNA studies.

### 2.9 Pre-analytical stability

Proper preservation of miRNAs during the preanalytical phase is essential to ensure reliable results. Several studies have shown that miRNAs remain stable for several months when stored under refrigerated or frozen conditions (4 °C, −20 °C, and −80 °C), whereas they undergo degradation when exposed to room temperature (Trakunram et al., 2019).

Among the many factors that influence miRNA stability, the following stand out: the type of sample (serum is usually more stable than plasma), the anticoagulants used in blood collection, and the GC content in the sequence (miRNAs with less than 40% are more susceptible to degradation) (Trakunram et al., 2019). However, it is also important to consider two additional aspects that are critical in miRNA analysis: the degree of hemolysis of the samples and the normalization methods used.

Hemolysis is a significant interfering factor, releasing cellular contents that contaminate the sample and alter circulating miRNA profiles. While visual inspection is commonly used to exclude grossly hemolyzed samples, several studies have demonstrated that low levels of hemolysis may not be visually detectable. Objective molecular approaches based on erythrocyte-enriched miRNAs, particularly the miR-451a/miR-23a-3p ratio, have been shown to be highly sensitive indicators of hemolysis and are recommended as quality control strategies in circulating miRNA studies (Shah et al., 2016).

Data normalization is another critical factor required to ensure comparable results across studies. Rather than relying on fixed endogenous reference miRNAs, current best practice recommends selecting normalization controls based on their stability under the specific experimental conditions of each study. This is most reliably achieved using large-scale miRNA profiling approaches, such as RNA sequencing or microarray platforms, which allow identification of the most stable miRNAs for use as endogenous controls in downstream targeted assays. This strategy reduces technical bias and improves reproducibility across studies (Hamam et al., 2017).

### 2.10 MiRNA analysis techniques

After miRNA extraction, it is essential to select a suitable analysis technique for miRNA detection and quantification to obtain reliable data. The main techniques currently available have distinctive characteristics that should be considered according to the objectives of the study.

#### 2.10.1 Quantitative polymerase chain reaction (qPCR)

This is a technique in which target miRNAs are reverse transcribed into complementary DNA (cDNA), which is used as a template for qPCR, with acceptable sensitivity and relative speed. Its advantages include the capacity for absolute quantification and the availability of standardized commercial kits. However, it has limitations in primer design due to the short length of miRNAs, susceptibility to contamination, and the requirement for high-purity samples. Although qPCR is not suitable for unbiased global discovery of completely novel miRNAs, cancer-specific and custom-designed RT-qPCR array panels enable targeted discovery and profiling of large predefined sets of miRNAs and have been widely used in biomarker-oriented studies (Ouyang et al., 2019).

#### 2.10.2 Digital PCR (dPCR)

Is an advanced quantitative technique that enables absolute miRNA quantification without the need for standard curves by partitioning the sample into thousands of individual reactions. This approach provides higher sensitivity and precision than conventional qPCR, particularly for low-abundance circulating miRNAs and small fold-change differences. In addition, dPCR is less affected by amplification efficiency and PCR inhibitors, which makes it especially suitable for validation of candidate miRNA biomarkers in biofluids. However, its higher cost, lower throughput, and limited multiplexing capacity currently restrict its application in large-scale screening studies. Nevertheless, dPCR represents a valuable tool for analytical validation and clinical translation of circulating miRNA biomarkers (Hindson et al., 2011; Borzi et al., 2017).

#### 2.10.3 RNA sequencing (RNA-seq)

Is a comprehensive technique that enables the identification of novel miRNAs and transcriptome-wide analysis. Although RNA-seq provides very high accuracy (∼99.99%), library preparation is complex, data analysis is time-consuming, and costs remain high, limiting its feasibility for routine clinical diagnostics. However, it is widely used in research to identify new potential biomarkers in different diseases (Ouyang et al., 2019).

#### 2.10.4 Microarrays

Are a biochip-based analysis platforms that enable rapid detection of miRNAs by immobilization of DNA probes on solid surfaces. Detection relies on the specific hybridization between immobilized probes and target miRNAs, generating signals that allow their quantification. This technology offers high throughput, enabling simultaneous analysis of multiple miRNAs and relatively rapid data generation. However, the specificity is moderate since hybridization efficiency may be reduced due to the short length of miRNA probes, which can hinder efficient target labeling, likewise, the low melting temperature can generate unspecific hybridizations, compromising the reliability of the results (Ouyang et al., 2019).

Comparative summary of the main techniques used for miRNA extraction and detection, including their characteristics for sensitivity, specificity, cost, and execution time. This table helps identify the most suitable methodologies for clinical diagnostic studies (e.g., qPCR) and research (e.g., RNA-seq) in breast cancer, emphasizing the importance of selection based on study objectives.

Understanding these methodologies and the variability in their application is paramount for interpreting the reliability and consistency of the reported miRNA biomarkers. These findings highlight the need to standardize analytical workflows, including the appropriate selection of extraction methods (Table 3) and strict control of pre-analytical conditions to ensure the reliability of clinical studies and research.

**TABLE 3:** Extraction and detection techniques for circulating miRNA analysis.

Technique category	Method	Sensitivity	Specificity	Cost	Advantages and limitations	Time of execution
Extraction	Trizol	High	Moderate	Moderate	Widely used and cost-effective RNA extraction method with good yield; however, it is labor-intensive and may co-isolate contaminants affecting miRNA purity	1–2 h
​	miRNeasy commercial kit	High	High	High	Provides standardized and reproducible RNA extraction with higher purity; however, higher cost may limit routine large-scale use	30–60 min
Detection	Quantitative Real-time PCR (qRT-PCR)	High	High	Moderate	Highly sensitive and widely used for targeted miRNA quantification; however, it is limited to the analysis of predefined miRNAs	2–4 h
​	Digital PCR (dPCR)	Very high	Very high	High	Enables absolute quantification with high sensitivity and precision; however, specialized equipment and higher costs limit accessibility	2–4 h
​	RNA-Seq	Very high	Very high	High	Allows comprehensive profiling and discovery of novel miRNAs; however, it is expensive, computationally demanding, and time-consuming	Days
​	Microarrays	Low-moderate	Low-moderate	Moderate-high	Enables simultaneous analysis of many known miRNAs; however, lower sensitivity and inability to detect novel miRNAs limit its utility	6–8 h

Abbreviations: miRNA, microRNA; qRT-PCR, quantitative reverse transcription polymerase chain reaction; dPCR, digital polymerase chain reaction; RNA-seq, RNA, sequencing.

## 3 Results

### 3.1 Quality assessment of included studies (QUADAS-2)

The methodological quality of the 16 included diagnostic studies was assessed using the QUADAS-2 tool, and the results are summarized in Table 1. Overall, most studies showed a high risk of bias in the patient selection domain, mainly due to case–control designs, small sample sizes, and non-consecutive recruitment. The index test domain was frequently judged as high or unclear risk, largely because thresholds were not specified and blinding to the reference standard was often not reported. In contrast, the reference standard domain was generally rated as low risk, as histopathological confirmation was consistently used. Flow and timing was frequently classified as unclear or high risk, mainly due to insufficient reporting on the time interval between sample collection and diagnosis or incomplete inclusion of participants in the final analysis.

## 4 MiRNAs used as biomarkers for early diagnosis in breast cancer

Considering the current knowledge about the biogenesis process and the different mechanisms of gene regulation mediated by miRNAs in breast cancer that have been previously mentioned, several studies have shown that miRNA expression varies significantly between patients diagnosed with breast cancer and healthy patients. The 16 studies analyzed identified different miRNAs with differential expression in breast cancer patients versus controls, with a focus on their early diagnostic potential.

### 4.1 Individual miRNAs with high diagnostic potential

Liu et al. performed a comprehensive bioinformatics analysis of genomic databases (GEO) and experimental validation by qRT-PCR, demonstrating that miR-21-5p showed significantly elevated expression in exosomes of breast cancer patients. miR-21-5p (such miRNA) showed high diagnostic potential, with an AUC of 0.961, sensitivity of 86.7% and specificity of 93.3%, highlighting its ability to distinguish between patients and healthy controls (Liu et al., 2021). Moreover, Qiao et al. analyzed samples from 51 breast cancer patients, 47 healthy controls and 45 patients with benign breast disease, identifying that miR-200c was downregulated in serum-derived exosomes from breast cancer patients and in breast cancer cell exosomes relative to controls, in cohort I, miR-200c was downregulated in serum exosomes from cancer patients. This miRNA reached an AUC of 0.854, outperforming traditional markers such as CEA, CA-125 and CA 15-3, suggesting its potential as a diagnostic biomarker (Qiao et al., 2024).

Other individual miRNAs also showed diagnostic value. Konoshenko et al. in exosome-enriched EVs from total blood from 43 breast cancer patients and 35 healthy individuals, observing that miR-92a is overexpressed in plasma exosomes of breast cancer patients, whereas miR-25-3p is more abundant in total blood exosomes, which included plasma and cell surface. Both miRNAs are involved in inducing angiogenesis, epithelial-mesenchymal transition (EMT) and metastatic dissemination, which favors cancer progression (Konoshenko et al., 2020). Wang et al. reported that miR-365-5p is significantly decreased in plasma exosomes of breast cancer patients (10 patients vs. 10 controls), with an AUC of 0.958, acts as a tumor suppressor by negatively regulating the PDGFB gene, involved in metastasis (Wang et al., 2021).

Similarly, Li et al., observed by qRT-PCR that miR-148a is under expressed in serum exosomes of 125 breast cancer patients compared with 50 patients with benign tumors and 40 healthy controls, showing an AUC of 0.897 to discriminate between malignant and controls and 0.806 to differentiate malignant from benign tumors. Furthermore, its decrease was associated with the promotion of lymph node metastasis and worse survival (Li et al., 2020).

In another study, focused on early diagnosis, Kim et al., evaluated miR-202 in plasma from 30 breast cancer patients and 30 controls by qRT-PCR, observing that miR-202 is significantly overexpressed in the group of patients with cancer. Receiver operating characteristic (ROC) analysis showed excellent diagnostic performance (AUC: 0.95, sensitivity: 90%, specificity: 93%) and it was determined that elevated levels of this miRNA were associated with a 9.5-fold increased risk of developing breast cancer (Kim et al., 2020).

Kan et al. extended the knowledge on exosomal miRNAs by studying miR-92b-5p in 59 patients and 53 controls by qRT-PCR. This miRNA showed progressive overexpression according to tumor stage (5.08-fold in stage I, 5.58-fold in stage II and 4.57-fold in stage III) in breast cancer patients and an AUC of 0.787. They demonstrated that exosomal miR-92b-5p suppresses the MTSS1L gene, a metastatic suppressor that modulates actin dynamics, and that exosomes enriched in miR-92b-5p increase cell migration *in vitro* (Kan et al., 2024).

However, it should be noted that many of the studies reporting very high AUC values were classified as having a high or unclear risk of bias in the QUADAS-2 assessment, particularly in the patient selection and index test domains (Table 1).

### 4.2 MiRNA panels for early diagnostics

In addition to individual miRNAs, several studies proposed the use of miRNA panels to improve diagnostic accuracy. Pan et al. identified a panel of 16 miRNAs (including miR-6806-5p, miR-1292-3p and miR-660-3p) in plasma exosomes by sequencing 56 blood samples from breast cancer patients and 40 healthy women. These miRNAs showed a differential expression profile and were associated with crucial pathways such as TGF-β signaling and DNA damage response. They developed a predictive model based on the miRNAs that achieved an accuracy of 97.22% in the training phase and 95.83% in the test phase, the AUC values obtained ranged from 0.936 to 0.979 outperforming conventional markers (Pan et al., 2022). Li et al. analyzed 400 plasma and 406 serum samples, identifying four miRNAs in plasma (miR-106a-3p, miR-106a-5p, miR-20b-5p and miR-92a-2-5p) and four in serum (miR-106a-5p, miR-19b-3p, miR-20b-5p and miR-92a-3p). A serum panel based on these miRNAs achieved an AUC of 0.974 in the test phase, with sensitivity between 87% and 94% (Li et al., 2018). Subsequently, the same group identified a set of five miRNAs (let-7b-5p, miR-122-5p, miR-146b-5p, miR-210-3p and miR-215-5p) overexpressed in plasma from 257 patients, whose panel showed an AUC of 0.978 in the validation phase (Li et al., 2019).

More recently, (Spychalski et al., 2025), proposed a novel diagnostic strategy based on the analysis of extracellular vesicle subpopulations selectively enriched for human epidermal growth factor receptor 2 (HER2) and cluster of differentiation 24 (CD24) in plasma from women with BI-RADS 4 breast lesions. Using immunomagnetic isolation combined with next-generation miRNA sequencing, the authors identified distinct and complementary EV-miRNA signatures associated with malignancy. Individual miRNAs achieved AUC values of up to 0.87, while a four-miRNA panel (miR-340-5p, miR-598-3p, miR-15b-5p, and miR-126-3p) reached an AUC of 0.95, with sensitivity of 87% and specificity of 91%, and was subsequently validated by qPCR. Importantly, the cohort was enriched for stage 0–I disease, highlighting the potential of subpopulation-resolved EV-miRNA panels for early breast cancer detection in clinically ambiguous BI-RADS 4 lesions.

Li et al. analyzed 60 samples from breast cancer patients and 20 healthy individuals by qRT-PCR and identified that miR-3662, miR-146a and miR-1290 had significantly elevated levels in serum exosomes of breast cancer patients. These miRNAs were associated with lymph node metastasis and advanced stages of disease, suggesting their usefulness not only for initial diagnosis but also for monitoring tumor progression (Li et al., 2021).

Asgari et al. They provided additional evidence on the expression of five miRNAs (miR-21, miR-155, miR-182, miR-373 and miR-126) by qRT-PCR in exosomes isolated from 7 serum samples of breast cancer patients and seven samples of healthy controls. They identified that four miRNAs were overexpressed in the cancer patient group miR-21, miR-155, miR-182 and miR-373. Their results support the diagnostic potential of miRNAs; however, it is important to mention that the small sample size affects the robustness of the results (Asgari and Rezaie, 2022).

### 4.3 Role of miRNAs in progression and metastasis with diagnostic implications

Some miRNAs identified in the studies reviewed, in addition to their diagnostic potential, also showed a role in breast cancer progression and metastasis.

The miRNAs miR-3662, miR-146a and miR-1290, identified by Li et al. (Yang et al., 2024), were associated with lymph node metastasis and advanced stages of disease, suggesting their usefulness not only for initial diagnosis but also for monitoring tumor progression. Yang et al. (Yang et al., 2024), proposed miR-6831-5p as a biomarker for TNBC, mainly in cases of chemoresistance. Through bioinformatics analysis, they revealed that miR-6831-5p modulates key pathways such as MAPK and Hippo signaling, which could help identify patients resistant to standard therapies and guide alternative treatments.

### 4.4 Discrepancies and challenges in the detection of diagnostic miRNAs

Importantly, not all studies showed conclusive results, underscoring the challenges in standardization and variability of biomarkers. Contradictory results were evidenced in the study by Gonzalez-Villasana et al. where miR-145, miR-155 and miR-382 showed no significant differences in exosomes from twenty patients versus five controls. The authors attributed this finding to the limited sample size and additionally, that many patients had received previous treatment, which demonstrates the need to standardize conditions in future research (Gonzalez-Villasana et al., 2019). However, the role of miR-155 as a biomarker was supported by Gao and collaborators, who demonstrated its overexpression in plasma and tumor cells of breast cancer patients by qRT-PCR, this miRNA is induced by by forkhead box P3 (FOXP3) through repression of breast cancer 1 (BRCA1), a tumor suppressor gene, its diagnostic capacity was moderate (AUC of 0.77 for localized cancer and 0.75 for metastatic) but its usefulness in early stages is still relevant (Gao B. et al., 2017).

## 5 Discussion

**FIGURE 3:**
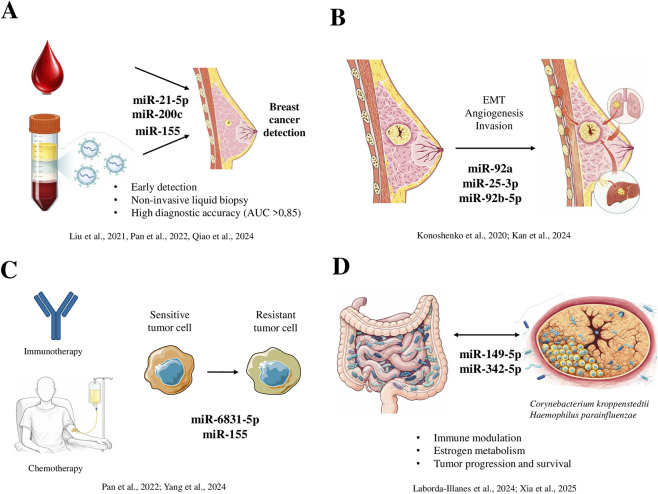
Overview of the biological and clinical roles of circulating microRNAs in breast cancer. Circulating microRNAs (miRNAs) represent non-invasive diagnostic biomarkers for early breast cancer detection and are associated with tumor progression and metastasis through processes including epithelial–mesenchymal transition (EMT), angiogenesis, and invasion. In addition, circulating miRNAs provide insight into therapeutic response and resistance. Emerging evidence further supports a microbiome–miRNA axis in breast cancer, whereby specific bacterial species are associated with dysregulated miRNAs involved in immune modulation, estrogen metabolism, tumor progression, and patient survival. **(A)** Circulating miRNAs as non-invasive diagnostic biomarkers **(B)** miRNA signatures associated with tumor progression and metastasis **(C)** miRNAs as indicators of therapy response and resistance **(D)** Microbiome-miRNA axis in breast cancer.

This review highlights the potential of circulating miRNAs as diagnostic biomarkers for breast cancer, demonstrating expression patterns that vary with tumor stage, metastatic status, and therapeutic response. Compared with mammography, the current gold standard that remains limited in younger women or those with dense breast tissue, and traditional serum biomarkers such as carcinoembryonic antigen (CEA), cancer antigen 125 (CA-125), and cancer antigen 15-3 (CA 15-3), miRNAs represent a non-invasive alternative that may offer superior diagnostic performance in certain contexts. For instance, (Liu et al., 2021; Qiao et al., 2024), reported high diagnostic accuracies for individual miRNAs such as miR-21-5p and miR-200c, with AUC values exceeding 0.85. Notably, Qiao et al. found that miR-200c outperformed conventional serum markers, suggesting that miRNAs may complement existing screening tools, particularly in early detection and cases where mammography yields inconclusive results. The diverse functional roles of miRNAs in breast cancer, including their diagnostic value, involvement in tumor progression and metastasis, regulation of therapy response, and interaction with the microbiome, are schematically summarized in Figure 3.

Despite these encouraging findings, several methodological issues limit their immediate clinical application. These limitations are reflected in the QUADAS-2 assessment, which identified frequent risks of bias related to patient selection, index test execution, and flow and timing across the included studies. Future diagnostic studies should prioritize treatment-naïve cohorts and standardized pre-analytical workflows to minimize treatment-related confounding and improve reproducibility across studies.

In addition to sample size limitations, substantial methodological heterogeneity was observed across the included studies, particularly in extracellular vesicle (EV) isolation protocols (Table 4) including ultracentrifugation, polymer-based precipitation kits (e.g., ExoQuick), membrane affinity columns, and commercial rapid extraction reagents (Gonzalez-Villasana et al., 2019). These approaches differ markedly in terms of vesicle recovery efficiency, co-isolation of non-exosomal components, and RNA yield, which can directly influence miRNA quantification and downstream diagnostic performance (Gonzalez-Villasana et al., 2019).

**TABLE 4:** Methodological heterogeneity of extracellular vesicle (EV) isolation methods and miRNA normalization strategies in included studies.

Study	Cohort (n)	BC stage/Subtype	Sample type	EV isolation	miRNA quantification	Normalization	Study design	Functional evidence
Exosomal hsa-miR-21-5p is a biomarker for breast cancer diagnosis (Liu et al., 2021)	30 BC/30 HC	NR (mixed stages implied; not explicitly stratified)	Plasma- derived exosomes	Differential ultracentrifugation	RT-qPCR	U6 snRNA	Case–control diagnostic study with bioinformatic screening and experimental validation	Yes
Serum exosomal miR-200c is a potential diagnostic biomarker for breast cancer (Qiao et al., 2024)	Cohort I: 51 BC/47 HC; Cohort II: 44 BC/30 HC/45 BBD	NR (treatment-naïve; stage and subtype not stratified)	Serum- derived exosomes	Polymer-based precipitation kit (Hieff® Quick Exosome Isolation Kit, Yeasen)	qRT-PCR (screening by miRNA deep sequencing in cell exosomes)	U6 snRNA	Case–control diagnostic study with two independent validation cohorts	Partial
Total blood exosomes in breast cancer: Potential role in crucial steps of tumorigenesis (Konoshenko et al., 2020)	43 BC/35 HC	Early–locally advanced BC; IDC; ER+/−, PR+/−, HER2^+/−^ (detailed clinicopathological data reported)	Plasma exosomes and total blood exosomes (plasma + blood cell–surface-associated)	Ultrafiltration followed by differential ultracentrifugation	qRT-PCR (TaqMan assays)	miR-16-5p (cel-miR-39 spike-in control)	Case–control study with extensive *in vitro* functional assays	Yes
A Breast Cancer Prediction Model Based on a Panel from Circulating Exosomal miRNAs (Pan et al., 2022)	56 BC/40 HC	Predominantly early-stage BC (T1–T2: 82.1%); ER+/−, PR+/−, HER2^+/−^	Plasma- derived exosomes	Differential ultracentrifugation (filtration + ultracentrifugation)	Small RNA sequencing (Illumina HiSeq4000)	Reads per million (RPM); DEGseq normalization	Case–control study with machine learning–based diagnostic models (LASSO, SVM, GBDT, Random Forest)	No
Circulating exosomal miR-363-5p inhibits lymph node metastasis (Wang et al., 2021)	Discovery cohort: 10 BC/10 HC; Validation using TCGA (n = 1044) and GEO GSE38167 (n = 31)	ER+/HER2−; stage I–II IDC or DCIS; LN + vs. LN− stratification	Plasma- derived exosomes	Differential ultracentrifugation (100 000 g × 2; filtration 0.22 µm)	Small RNA sequencing (Illumina HiSeq) + qRT-PCR validation	TPM normalization (RNA-seq); U6 for qRT-PCR	Case–control study with lymph node stratification and multi-cohort validation	Yes – *in vitro* functional assays and target validation (PDGFB)
Detection significance of miR-3662, miR-146a, and miR-1290 in serum exosomes (Li et al., 2021)	60 BC/20 HC	Stage I–III; subtype not specified; LN + vs. LN− stratification	Serum- derived exosomes	Polymer-based precipitation (Exosome Isolation Reagent, RiboBio)	qRT-PCR	2^−ΔΔCT^; endogenous control not clearly specified	Case–control study with longitudinal monitoring (pre-/post-surgery and chemotherapy)	No
Identification of serum exosomal miR-148a as a novel prognostic biomarker (Li et al., 2020)	125 BC/50 Benign/40 HC	TNM I–IV; subtype NR	Serum exosomes	ExoQuick precipitation (System Biosciences)	qRT-PCR	cel-miR-39 spike-in	Case–control; prognostic follow-up	No
Diagnostic value of circulating miR-202 in early-stage breast cancer (Kim et al., 2020)	30 BC/30 HC	TNM I–III; subtype NR	Plasma	Not applicable (circulating miRNA)	RT-qPCR (TaqMan assays)	miR-16 (endogenous control)	Case–control; diagnostic accuracy study	No
A five-miRNA panel in plasma was identified for breast cancer diagnosis (Li et al., 2019)	257 BC/257 HC	TNM in situ–III; luminal, HER2-enriched, TNBC	Plasma	ExoQuick precipitation (System Biosciences) for exosome analysis only	qRT-PCR (Bulge-Loop miRNA qRT-PCR; Exiqon panel in screening phase)	cel-miR-39 + miR-16 (plasma/exosomes); RNU6B for tissue	Multiphase case–control study (screening, training, testing, external validation)	No
Presence of circulating miR-145, miR-155, and miR-382 in serum exosomes (Gonzalez-Villasana et al., 2019)	20 BC/5 HC	Stage I–IV (ductal carcinoma)	Serum	ExoQuick precipitation	qRT-PCR (SYBR Green)	miR-16 (endogenous); cel-miR-39 tested	Case–control, exploratory	Indirect (literature-based)
Circulating microRNAs from the miR-106a–363 cluster (Li et al., 2018)	Plasma: 200 BC/200 HC; Serum: 204 BC/202 HC; Exosomes: 32 BC/32 HC	In situ–III; luminal, HER2-enriched, TNBC	Plasma, serum; plasma- and serum-derived exosomes	ExoQuick precipitation	qRT-PCR (SYBR Green)	Plasma/exosomes: cel-miR-39 + miR-16; Serum/exosomes: cel-miR-39 + miR-1228; Tissue: U6	Multiphase case–control (training, testing, external validation)	Indirect (tissue correlation + pathway analysis)
MicroRNA-155, induced by FOXP3 through transcriptional repression of BRCA1, is associated with tumor initiation in human breast cancer (Gao et al., 2017b)	Plasma cohort: total n = 259 (BC, benign, family history, controls); Exosome analysis: *in vitro* only	DCIS, early-stage, localized, metastatic; enrichment in ER−/PR− and TNBC	Plasma; blood cells; cell culture–derived exosomes	Ultracentrifugation (cell culture medium)	Nested qRT-PCR (plasma/exosomes); TaqMan qPCR (cells)	cel-miR-39 (plasma/exosomes), RNU6B (cells)	Case–control (human cohorts) + mechanistic *in vitro* study	Yes (FOXP3–BRCA1–miR-155 axis)
Differential Expression of Serum Exosomal miRNAs in Breast Cancer Patients and Healthy Controls (Asgari and Rezaie, 2022)	7 BC/7 HC	Primary BC; mixed grades (I–III); ER+/PR+/HER2+ reported	Serum	ExoQuick precipitation kit	qRT-PCR	Snord47	Case–control (pilot study)	No
Correlative expression of exosomal miRNAs in chemotherapy resistance of TNBC (Yang et al., 2024)	TNBC: n = 36 (Sequencing: 3 sensitive vs. 3 resistant; Validation: 15 sensitive vs. 15 resistant)	Triple-negative breast cancer; chemotherapy-sensitive vs. resistant (AC-sequential T)	Serum	Commercial rapid exosome extraction kit (YEASEN)	Small RNA sequencing + qRT-PCR validation	Not clearly specified (2^−ΔΔCT^ used)	Observational, case–control (chemosensitivity)	No
Exosomal microRNA-92b is a diagnostic biomarker in breast cancer (Kan et al., 2024)	59 BC/53 HC	Stage I–III; mixed subtypes (incl. TNBC, ER+, HER2+)	Serum	ExoQuick Exosome Precipitation Solution	miRNA microarray screening + RT-qPCR	U6 snRNA	Case–control diagnostic study with mechanistic validation	Yes (*in vitro* functional assays and target validation)
miRNA panel from HER2+ and CD24^+^ plasma extracellular vesicle subpopulations as biomarkers of early-stage breast cancer (Spychalski et al., 2025)	113 (27 malignant; 86 benign BI-RADS 4)	Early-stage BC (stage 0–I); DCIS and invasive; HER2 variable	Plasma	Immunomagnetic isolation (TENPO; HER2+ and CD24^+^ EV subpopulations)	RNA sequencing (NGS) with qRT-PCR validation	RUVSeq/DESeq2 (RNA-seq); not specified for qRT-PCR	Prospective diagnostic accuracy study	Yes

Abbreviations: miRNA, microRNA; EV, extracellular vesicle; BC, breast cancer; HC, healthy control; BBD, benign breast disease; qRT-PCR, quantitative reverse transcription polymerase chain reaction; RNA-seq, RNA, sequencing; RPM, reads per million; TPM, transcripts per million; snRNA, small nuclear RNA; TNBC, triple-negative breast cancer; IDC, invasive ductal carcinoma; DCIS, ductal carcinoma *in situ*; LN, lymph node; ER, estrogen receptor; PR, progesterone receptor; HER2, human epidermal growth factor receptor 2; NGS, next-generation sequencing; RNU6B, RNA U6 small nuclear 2.

Likewise, considerable variability was found in the selection of internal controls and normalization strategies, which were not consistently reported across studies. The included reports used endogenous miRNAs (e.g., miR-16, miR-191), small nuclear RNAs (U6, Snord47), and exogenous spike-in controls such as cel-miR-39, either alone or in combination. Each strategy has inherent limitations, as endogenous miRNAs may be dysregulated in cancer, while spike-in controls do not account for biological variability during RNA extraction. This lack of standardization likely contributes to inter-study variability in reported expression levels and AUC values, complicating direct comparisons and meta-analytic synthesis.

Therefore, the diagnostic accuracy of circulating and EV-derived miRNAs should be interpreted in light of these pre-analytical and analytical differences. Future studies should prioritize standardized extracellular vesicles isolation protocols and consensus normalization strategies to improve reproducibility and facilitate clinical translation.

In addition, efforts toward methodological standardization are increasingly supported by international guidelines aimed at improving reproducibility in extracellular vesicle research. Standardized protocols for sample collection, vesicle isolation, RNA extraction, and miRNA quantification are essential to minimize technical variability between laboratories. Adherence to the experimental and reporting recommendations proposed by the International Society for Extracellular Vesicles (MISEV guidelines) may facilitate cross-study comparisons and improve the reliability of EV-associated miRNA measurements (Théry et al., 2018). The implementation of harmonized protocols and multicenter validation studies will be critical to ensure that circulating and EV-derived miRNA biomarkers can be reproducibly translated into clinical practice.

Gonzalez-Villasana et al. (2019) demonstrated that the expression of miR-145, miR-155, and miR-382 varied significantly depending on cohort size and pretreatment status, highlighting how pre-analytical variability undermines reproducibility.

Beyond these concerns, the marked molecular heterogeneity of breast cancer—including luminal tumors characterised by estrogen and progesterone receptor expression (ER/PR-positive), human HER2+, and TNBC—raises the question of whether miRNA signatures represent universal biomarkers or are instead subtype-specific (Li et al., 2018). Importantly, heterogeneity also exists within HER2-positive disease itself. HER2-positive tumors may be either hormone receptor–positive (HR+/HER2+), often referred to as triple-positive breast cancer, or hormone receptor–negative (HR−/HER2+), subgroups that differ in molecular signalling pathways, therapeutic responsiveness, and clinical outcomes. Such biological diversity is likely to influence circulating miRNA expression profiles and may partly account for the variability in diagnostic performance reported across studies evaluating circulating or extracellular vesicle (EV)-associated miRNAs. Future studies should therefore incorporate stratification by molecular subtype to more precisely define subtype-specific circulating miRNA signatures.

Recent evidence further supports the relevance of subtype-specific interpretation of EV-derived miRNAs. A large prospective study published in 2025 evaluated serum extracellular vesicle–associated miR-21 in a well-characterized cohort of breast cancer patients and healthy donors, reporting significantly higher miR-21 levels in patients with active disease, particularly within the HER2+ subtype (Omarini et al., 2025). Notably, no significant differences were observed in TNBC, underscoring that the diagnostic utility of individual miRNAs may vary substantially across molecular subtypes. These findings reinforce the need to consider tumor biology and receptor status when evaluating the clinical applicability of circulating miRNA biomarkers and may partly explain the heterogeneity in diagnostic performance reported across studies.

Further supporting the concept of subtype- and compartment-specific EV-derived miRNA signatures, a recent prospective study by (Spychalski et al., 2025) evaluated plasma extracellular vesicle subpopulations selectively enriched for HER2 and CD24 in women with Breast Imaging Reporting and Data System (BI-RADS) 4 breast lesions. By combining immunomagnetic isolation of HER2+ and CD24^+^ EVs with next-generation miRNA sequencing, the authors identified distinct and complementary EV-miRNA profiles associated with malignancy, achieving an AUC of up to 0.87 for individual miRNAs and 0.95 for a four-miRNA EV-based panel validated by qPCR. Importantly, the study population was enriched for stage 0–I disease, underscoring the potential of subpopulation-resolved EV-miRNA approaches for early breast cancer detection in clinically challenging settings such as BI-RADS 4 lesions. While the specialized isolation platform limits direct methodological comparability with other studies, these findings highlight how targeting biologically defined EV subfractions may enhance diagnostic accuracy beyond total EV or free-circulating miRNA analyses.

In addition, differences in RNA extraction methods, normalization strategies, and data analysis pipelines further complicate cross-study comparisons. These technical and biological sources of variability must be addressed through standardized protocols and large-scale multicenter validation before miRNAs can be routinely adopted in clinical practice.

Complementary mechanistic insight is provided by recent preclinical evidence using humanized tumor mouse models, which allows controlled evaluation of circulating and EV–associated miRNAs under human-like tumor and immune conditions. In a proof-of-principle study, (Chen et al., 2025) demonstrated that both free-circulating and EV-derived human miRNAs can be detected in serum and dynamically modulated by breast cancer subtype (HER2+ vs. TNBC) and treatment exposure, including irradiation and immune checkpoint inhibition. Importantly, several miRNAs were selectively enriched in EVs compared with total serum, supporting the concept that EV-associated miRNAs represent a biologically distinct and potentially more specific biomarker compartment. Although derived from a preclinical model and therefore not included among diagnostic accuracy studies, these findings provide mechanistic support for the subtype-specific and compartment-dependent behavior of circulating miRNAs observed in clinical cohorts.

Importantly, miRNAs are not limited to diagnostic utility but are also involved in critical tumor processes with prognostic and therapeutic implications. Dysregulated expression of miRNAs such as miR-92a, miR-25-3p (Konoshenko et al., 2020) and miR-92b-5p (Kan et al., 2024) has been linked to angiogenesis and metastatic progression, while (Yang et al., 2024) identified miR-6831-5p as a regulator of chemoresistance in triple-negative breast cancer. These findings position miRNAs not only as biomarkers but also as potential therapeutic targets, with the capacity to inform personalized treatment strategies in aggressive disease subtypes.

While this review is primarily focused on the diagnostic and prognostic potential of circulating miRNAs, emerging evidence indicates that miRNA deregulation in breast cancer cannot be fully understood without considering the influence of the microbiome. Increasing data support the existence of a microbiome–miRNA axis in which both gut and tumor-associated microbial communities modulate host immune responses, estrogen metabolism, and therapeutic efficacy, thereby shaping tumor behavior and disease progression (Xia et al., 2025). Importantly, this interaction is bidirectional: microbial-derived metabolites and bacterial components can alter host miRNA expression profiles, while miRNAs may, in turn, influence microbial composition and function within the tumor microenvironment.

In this context, the study by (Laborda-Illanes et al., 2024) provides one of the most comprehensive integrative analyses to date, demonstrating that specific intratumoral bacteria enriched in metastatic breast cancer (such as *Corynebacterium kroppenstedtii* and *Haemophilus parainfluenzae*) are positively associated with the overexpression of metastasis-related miRNAs, including miR-149-5p and miR-342-5p. Notably, the combined microbial–miRNA signature identified in that study showed strong prognostic value for metastasis development and overall survival, highlighting the biological relevance of microbiota-driven miRNA modulation in breast cancer progression.

Although these observations are derived from tumor tissue, they have direct implications for the interpretation and clinical utility of circulating miRNAs. Tumor–microbiota interactions that shape intratumoral miRNA expression are likely to be reflected, at least in part, in circulating miRNA profiles, reinforcing their value as dynamic and integrative biomarkers of tumor biology. From a translational perspective, these findings raise the possibility that targeting microbial dysbiosis (through dietary, probiotic, or pharmacological interventions) could indirectly influence oncogenic miRNA programs. Although still largely supported by preclinical and associative evidence, such approaches may ultimately contribute to improved responses to systemic therapies, including immunotherapy, in selected patient subgroups (Xia et al., 2025). Therefore, incorporation of the microbiome–miRNA axis adds an important mechanistic layer to the understanding of miRNA-based biomarkers and opens new avenues for precision strategies in breast cancer management.

Beyond miRNA-based biomarkers, other molecular markers have also demonstrated diagnostic and prognostic relevance in breast cancer. For example, Wang et al. identified the tubulin alpha-1b chain (TUBA1B) as a prognosis- and immune-related biomarker, with experimental validation in breast cancer tissues and cell lines, showing moderate to high diagnostic accuracy and significant associations with tumor immunity and survival outcomes (Wang et al., 2024). Similarly, Aimaiti et al. reported that bystin (BYSL) is overexpressed in breast cancer and functions as an independent prognostic and immune-related biomarker, supported by extensive bioinformatic analyses and experimental validation (Aimaiti et al., 2025). Together, these observations suggest that miRNA-based assays should be interpreted within a broader biomarker landscape, and that future diagnostic strategies may benefit from integrative approaches combining circulating miRNAs with protein-based and immune-related biomarkers.

Nevertheless, most available studies rely on relatively small cohorts (Gao B. et al., 2017; Li et al., 2020; 2021; Asgari and Rezaie, 2022), which limits the generalizability of their findings. Small sample sizes are a well-recognized source of bias in diagnostic accuracy studies and may artificially inflate performance estimates, particularly the area under the ROC curve (AUC), due to overfitting and increased statistical variance (Gao S. et al., 2017). Studies including fewer than 30–50 participants per group are especially prone to reporting optimistic sensitivity and specificity values that are unlikely to be reproduced in independent populations.

This phenomenon is reflected in the QUADAS-2 assessment, where a high or unclear risk of bias was frequently observed in the patient selection and flow and timing domains, often due to limited sample sizes, non-consecutive recruitment, or lack of independent validation cohorts. These methodological limitations likely contribute to the exceptionally high AUC values (>0.95) reported in some individual studies included in this review. Therefore, such results should be interpreted with caution until confirmed in larger, prospective, and multicenter studies.

Larger, prospective studies are urgently needed to validate candidate miRNAs and, more importantly, to establish clinically robust multi-miRNA panels. Panels proposed by (Pan et al., 2022; Li et al., 2019) and more recently by Jing et al. (2024), who reported a five-miRNA serum panel (miR-10b-5p, miR-133a-3p, miR-195-5p, miR-195-3p, and miR-155-3p) for breast cancer detection, achieving an AUC of 0.948 in a validation cohort that included both screening and independent testing phases (Jing et al., 2024), have already demonstrated superior diagnostic accuracy compared with individual markers, underscoring the value of combinatorial approaches. In parallel, integrating miRNA testing with imaging and other molecular assays could further enhance sensitivity and specificity, while the minimally invasive nature of blood-based assays offers clear advantages for longitudinal monitoring.

Clinical translation of circulating miRNA biomarkers is still ongoing. A search of ClinicalTrials.gov using the keywords “microRNA” and “breast cancer” identified 21 registered clinical studies evaluating circulating or exosomal miRNAs in breast cancer patients. Most of these studies are observational in design, with only a small number of interventional trials, and most are currently recruiting, completed without published results, or of unknown status. Only one study has publicly available results to date, highlighting the limited availability of high-level clinical evidence. These trials primarily investigate the role of circulating miRNAs in early detection, prognosis, treatment response, and disease monitoring, underscoring the growing interest in miRNA-based liquid biopsies. However, the lack of reported results and heterogeneity in study designs indicate that clinical validation remains incomplete, and robust conclusions regarding clinical utility cannot yet be drawn.

A limitation of this review is the heterogeneity in the definition of early breast cancer across studies, as several investigations included mixed-stage populations or did not report AJCC/TNM staging, which may influence the interpretation of diagnostic performance.

From a clinical implementation perspective, the potential incorporation of circulating miRNA-based assays into current breast cancer screening pathways must also be evaluated in terms of cost-effectiveness. While mammography, ultrasound, and magnetic resonance imaging (MRI) remain the standard screening tools, they are associated with limitations related to cost, accessibility, radiation exposure, and reduced sensitivity in dense breast tissue. Blood-based miRNA assays could offer a complementary, minimally invasive, and potentially cost-efficient strategy, particularly for risk stratification and longitudinal monitoring. However, formal health economic evaluations comparing miRNA-based testing with existing screening modalities are currently lacking and should be addressed in future studies.

In conclusion, circulating and EV-derived miRNAs hold strong promise as biomarkers for the early diagnosis, prognosis, and treatment stratification of breast cancer. However, translating this promise into clinical practice requires overcoming significant barriers, including technical standardization, validation in diverse populations, and demonstration of cost-effectiveness within existing screening frameworks. Addressing these challenges will be critical for miRNAs to evolve from promising research tools into routine components of precision oncology.

## Data Availability

The original contributions presented in the study are included in the article/supplementary material, further inquiries can be directed to the corresponding authors.
